# All*-trans* retinoic acid and arsenic trioxide fail to derepress the monocytic differentiation driver Irf8 in acute promyelocytic leukemia cells

**DOI:** 10.1038/cddis.2017.197

**Published:** 2017-05-11

**Authors:** XiangZhen Liu, Juan Chen, ShanHe Yu, Li Yan, HeZhou Guo, JianMin Dai, Wu Zhang, Jiang Zhu

**Affiliations:** 1 State Key Laboratory for Medical Genomics, Shanghai Institute of Hematology and Collaborative Innovation Center of Hematology, Rui-Jin Hospital Affiliated to Shanghai Jiao Tong University School of Medicine, Shanghai, China; 2 School of Life Sciences and Biotechnology, Shanghai Jiao Tong University, Shanghai, China; 3 Collaborative Innovation Center of Systems Biomedicine, Shanghai, China

## Abstract

All-*trans* retinoic acid (ATRA) and/or arsenic trioxide (ATO) administration leads to granulocytic maturation and/or apoptosis of acute promyelocytic leukemia (APL) cells mainly by targeting promyelocytic leukemia/retinoic acid receptor alpha (PML/RAR*α*). Yet, ~10–15% of APL patients are not cured by ATRA- and ATO-based therapies, and a potential failure of ATRA and ATO in completely reversing PML/RAR*α*-driven oncogenic alterations has not been comprehensively examined. Here we characterized the *in vivo* primary responses of dysregulated genes in APL cells treated with ATRA and ATO using a GFP-labeled APL model. Although induced granulocytic differentiation of APL cells was evident after ATRA or ATO administration, the expression of the majority of dysregulated genes in the c-Kit^+^ APL progenitors was not consistently corrected. *Irf8*, whose expression increased along with spontaneous differentiation of the APL progenitors *in vivo*, represented such a PML/RAR*α*-dysregulated gene that was refractory to ATRA/ATO signaling. Interestingly, *Irf8* induction, but not its knockdown, decreased APL leukemogenic potential through driving monocytic maturation. Thus, we reveal that certain PML/RAR*α*-dysregulated genes that are refractory to ATRA/ATO signaling are potentially crucial regulators of the immature status and leukemogenic potential of APL cells, which can be exploited for the development of new therapeutic strategies for ATRA/ATO-resistant APL cases.

Introductions of all-*trans* retinoid acid (ATRA) and arsenic trioxide (ATO) have greatly improved the therapeutic outcomes of acute promyelocytic leukemia (APL), originally a highly fatal subtype of acute myeloid leukemia (AML), being regarded as a clinical paradigm of oncoprotein-targeted therapy.^1, 2, 3, 4^ Initial studies have revealed that APL-specific fusion protein promyelocytic leukemia/retinoic receptor alpha (PML/RAR*α*) fuels leukemogenesis largely by functioning as a strong transcriptional repressor that blocks the transcription of numerous myeloid differentiation-related genes, whereas ATRA at pharmacological dose converts PML/RAR*α* into a transcriptional activator upon its recognizing the ligand-binding pocket of RAR*α*, thereby releasing the differentiation arrest of APL cells to decrease their leukemic malignancy.^5, 6^ This theory has gained ongoing support from subsequent studies that have shown a previously unappreciated complexity in the actions of multiple layers of chromatin modifications.^7, 8, 9, 10^ On the other hand, numerous studies have also correlated ATRA- or ATO-induced PML/RAR*α* degradation with the clinical cure.^1, 11^ Mechanistically, ATO recognizes the N-terminal PML moiety to crosslink PML/RAR*α* molecules, which in turn renders PML/RAR*α* susceptible to a sumoylation/ubiquitination-coupled degradation mechanism that is active in nucleus,^12, 13^ Theoretically, the degradation of PML/RAR*α* not only diminishes its suppression on the transcriptions of crucial myeloid differentiation-related genes but also allows the restoration of the structure and function of other PML/RAR*α* action sites such as the PML nuclear body and TGF*β* signaling pathway that are crucial factors controlling the proliferation, survival and differentiation of hematopoietic cells.^11, 14, 15^ Nevertheless, whether ATRA-induced degradation of PML/RAR*α* is required for relieving APL cell-associated differentiation arrest remains controversial,^16, 17, 18^ as a moderate PML/RAR*α* degradation-promoting effect might occur only after the ATRA-bound PML/RAR*α* has accomplished its action of activating the transcription of the target genes originally repressed by the ligand-free PML/RAR*α*.^19^ Moreover, both ATRA and ATO exert a few PML/RAR*α*-independent regulatory effects that contribute to the restriction of APL malignancy.^20^

The exact cellular and molecular mechanisms underlying the therapeutic responses of APL cells to ATRA or ATO remain incompletely understood, especially in the *in vivo* setting. Relevantly, ~5–6% of human APL cases did not achieve complete clinical remission after receiving ATRA- and ATO-based treatments,^3, 21^ and another 5–10% of APL patients relapsed from complete clinical remission. The underlying mechanisms were uncovered only in a small portion of these primarily refractory or relapsed cases (i.e., the detection of specific mutations that undermined the specific binding of PML/RAR*α* by ATO or ATRA).^2, 21^ Therefore, no specialized therapeutic strategies have been developed for these refractory or relapsed cases.

The therapeutic resistance is most likely rooted in the inability of ATRA or ATO to correct all crucial oncogenic alterations emanating from PML/RAR*α*. For example, only in ~10% of PML/RAR*α*-target genes, their expressions were significantly altered after exposure to ATRA.^10^ In fact, how the expression of PML/RAR*α* target genes was restored after ATO treatment remains largely unexplored. In this study, we examined in a global manner how the dysregulated genes of APL cells responded to ATRA or/and ATO treatment *in vivo*. We have identified certain key oncogenic alterations that contribute to the maintenance of APL malignancy but are not corrected by ATRA or/and ATO treatment. The nodal factors central to these ATRA/ATO signaling-unresponsive programs represent the potential new therapeutic targets for APL, especially for those that are primarily refractory to or tend to relapse after receiving ATRA- and/or ATO-based therapies.

## Results

### APL cells respond to ATRA or ATO treatment *in vivo* by undergoing granulocytic differentiation and cell death

**Figure 1:**
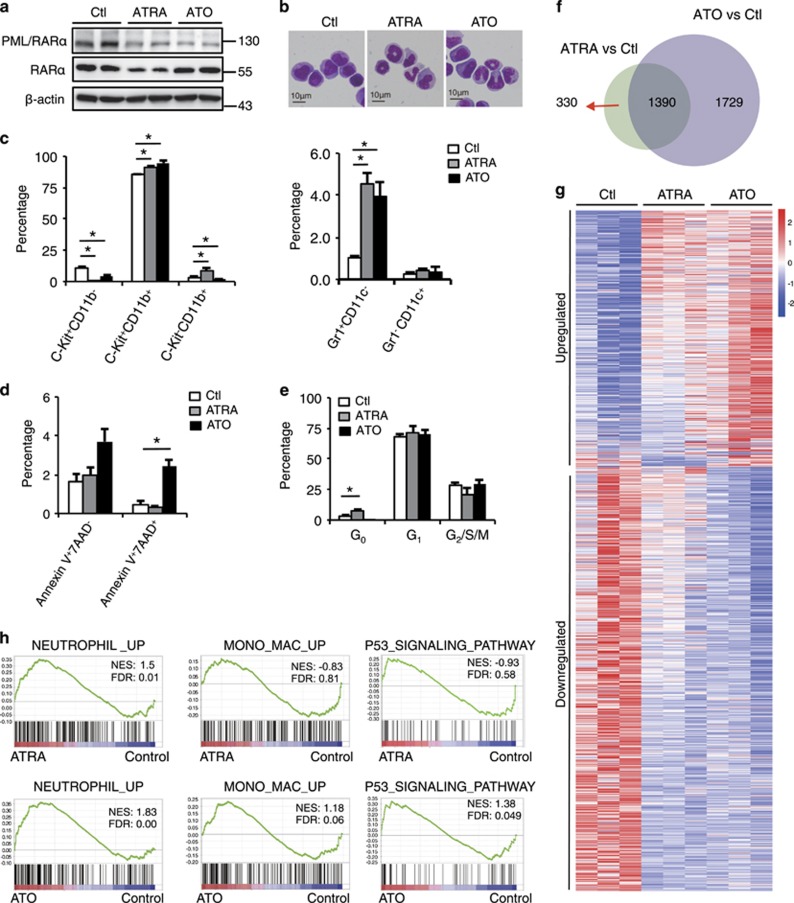
Global gene expression alterations in APL cells after ATRA or ATO treatment *in vivo*. (**a**-**e**) FVB/NJ mice were each injected intravenously with 1 × 10^6^ GFP^+^ syngeneic APL BM cells. At the overt leukemia phase, the recipients were treated with or without daily intraperitoneal injection of ATRA (10 mg/kg) or ATO (4 mg/kg) for 6 days, and the BM GFP^+^ APL cells from each group were sorted and analyzed. (**a**) Western blotting assay for RAR*α*and PML/RAR*α* protein levels using anti-RAR*α* and anti-PML antibodies. (**b**) Microscopic inspection of the sorted APL cells with Wright–Giemsa staining. (**c–e**) Statistic results of flow cytometry analyses of the expressions of c-Kit, CD11b, Gr-1 and CD11c for myeloid differentiation (**c**), Annexin V and 7AAD for cell survival (**d**), and HO33342 and Ki67 for cell cycle (**e**). (**f**) RNA sequencing showing the numbers and overlap of the differentially expressed (DE) genes between the ATRA-treated APL cells *versus* the control group and the ATO-treated APL cells *versus* the control group (*P*<0.05, fold change ≥1.5 and *n*=3). (**g**) Heatmap of the total DE genes altered by ATRA or ATO treatment. (**h**) GSEA analysis of the *in vivo* effects of ATRA or ATO on APL cells. The gene sets of neutrophil-associated upregulated, monocyte/macrophage-associated upregulated and P53 signaling pathway signatures were used, and the expression profiles of ATRA-treated *versus* control APL cell were shown in the upper panel, whereas the ATO-treated *versus* control APL cell were shown in the bottom panel. All data in this figure are presented as the mean±S.D., *P <0.05, **P<0.01

Previous studies on the therapeutic responses-mediating mechanisms of APL cells to ATRA or ATO were largely based on analyses of PML/RAR*α*-expressing leukemia cell lines following *in vitro* treatment.^7, 10^ To investigate how APL cells respond to ATRA or ATO *in vivo*, we labeled the murine APL bone marrow (BM) cells derived from *hMRP8-PML/RARα* transgenic mice (FVB/NJ) with GFP-expressing retroviral vector MigR1.^22^ This labeling did not alter APL cells’ *in vivo* repopulation capacity, morphology and immunophenotype (Supplementary Figure S1a; data not shown). Syngeneic recipients repopulated with GFP^+^ APL cells were treated with or without ATRA or ATO for 6 days, and GFP^+^ APL cells within the BM were collected for RNA sequencing and other analyses. In agreement with the data from the previous studies,^12, 23^ Both ATRA and ATO reduced PML/RAR*α* level, whereas ATRA but not ATO reduced RAR*α* level (Figure 1a). Both ATRA and ATO resulted in differentiation of APL cells as evidenced by morphological alterations (Figure 1b). Flow cytometry analyses showed that ATRA or ATO treatment for 6 days resulted in a partial myeloid differentiation as indicated by elevated CD11b expression, and a mild c-Kit reduction was detected following ATRA treatment (Figure 1c, left panel; Supplementary Figure S1b, upper panel). Interestingly, both ATRA and ATO also mildly induced the expression of granulocytic lineage marker Gr-1 but not that of monocytic/dendritic lineage marker CD11c of the CD11b^+^ APL sections (Figure 1c, right panel; Supplementary Figure S1b, bottom panel). ATO inhibited cell survival, whereas ATRA inhibited cell cycle of APL cells (Figures 1d and e; Supplementary Figures S1c and d).

RNA sequencing revealed that ATRA and ATO signaling significantly altered the mRNA levels of 1720 and 3119 genes, respectively (fold change >1.5, *P*<0.05; Figures 1f and g). Interestingly, heatmap showed that the gene sets whose expressions were altered by ATO highly overlapped with those altered by ATRA, but with greater alterations in ATO (Figure 1g). In accordance, the Kyoto Encyclopedia of Genes and Genomes analyses indicated that modulations of a number of pathways were shared by ATRA and ATO, including activation of the p53 signaling and MAPK pathways (Supplementary Figure S1e). In accordance with the preferential induction of Gr-1 by ATRA or ATO (Figure 1c, right panel), the Gene Set Enrichment Analysis (GSEA) showed that both ATRA and ATO promoted the differentiation of APL cells towards granulocytes rather than monocytes (Figure 1h). On the other hand, GSEA indicated that ATO but not ATRA significantly altered activation of the p53 signaling pathway (Figure 1h). These results indicate that APL cells respond to ATRA or ATO treatment *in vivo* primarily by undergoing granulocytic differentiation and cell death.

### The majority of the dysregulated genes in immature APL cells are refractory to the modulatory effects of ATRA and ATO

**Figure 2:**
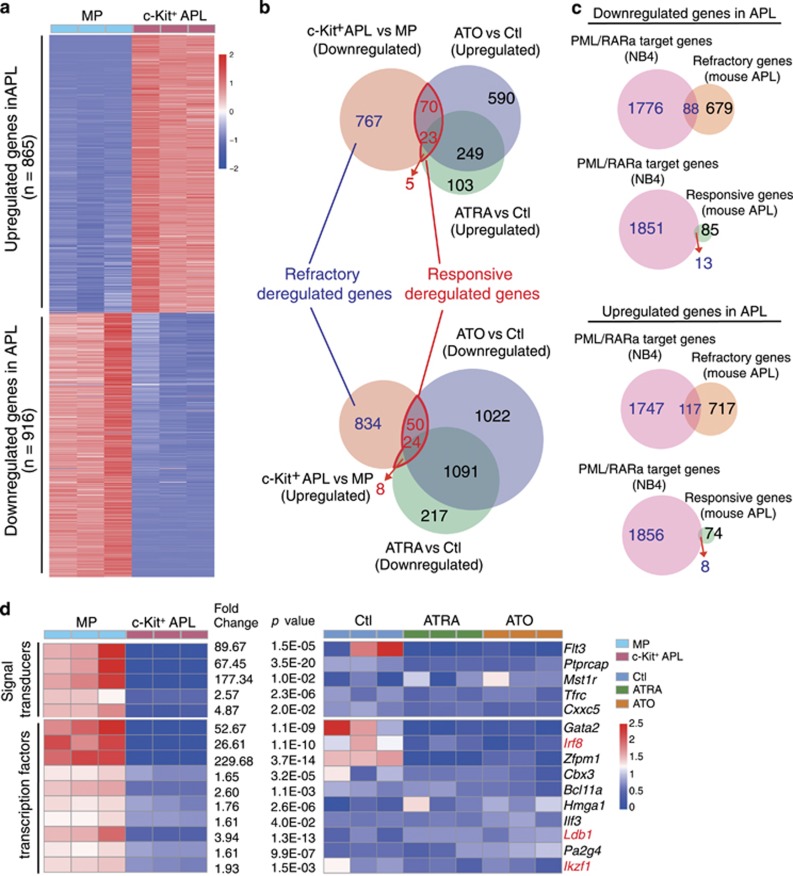
The majority of dysregulated genes in the APL progenitors are refractory to the corrective effect of ATRA or ATO. (**a**) Heatmap showing the dysregulated genes in the c-Kit^+^ APL progenitors compared with normal MPs (*P*<0.05, fold change ≥1.5). (**b**) Overlap analyses of ATRA/ ATO-refractory dysregulated genes with PML/RAR*α* target genes. (**c**) Overlap analyses of ATRA/ATO-responsive dysregulated genes with PML/RAR*α* target genes. (**d**) Heatmap showing the ATRA /ATO-refractory downregulated signal transducers and transcription factors

Previous studies have characterized the key ATRA/ATO-induced corrections that mediate the granulocytic differentiation or cell death. Nevertheless, the extent to which ATRA or ATO corrects the dysregulated gene pool in APL progenitors remains largely unexplored. In this regard, the identity for the cells of origin of APL remains undetermined, and they might originate from multiple stages of myeloid progenitors (MPs), including granulocytic and monocytic precursor (GMP), common myeloid precursor (CMP), and even megakaryotic and erythroid precursor (MEP).^24, 25, 26^ To make this issue more complicated, the leukemia-initiating cells (LICs) were recently shown to reside at more than one differentiation stages in all subtypes of human AML.^27^ Therefore, in a comprehensive manner, we first delineated a pool of genes that were differentially expressed between all c-Kit^+^ APL progenitor cells and the normal BM c-Kit^+^Lin^−^Sca-1^−^ subpopulation that are enriched with multiple types of MPs, including CMP, GMP, MEP, monocytic progenitor and granulocytic progenitor,^28^ and assumed the differentially expressed genes as a likely candidate 1pool of dysregulated genes in immature APL cells. As shown in Figure 2a, the expressions of 916 genes were downregulated, whereas those of 865 genes were upregulated in the immature c-Kit^+^ APL progenitors (fold change >1.5, *P*<0.05). Surprisingly, the overlap analyses between the ATRA/ATO-responsive genes and the dysregulated genes showed that only ~10% of these potentially dysregulated genes were ATRA/ATO-responsive genes, leaving about 90% of them basically refractory to ATRA or/and ATO signaling (Figure 2b). Reference to a well-characterized PML/RAR*α*-target gene pool that was identified in human APL cells revealed that 101 potentially downregulated genes in the APL progenitors were the direct target genes of PML/RAR*α*.^10^ Similarly, only ~10% of these genes (13 out of 101) were significantly upregulated by ATRA or ATO, whereas 88 were not (Figure 2c, upper panel). In a similar rate, of 125 potentially upregulated PML/RAR*α*-target genes in the APL progenitors, 8 were downregulated by ATRA or ATO, whereas 117 were not (Figure 2c, bottom panel).

Next, we focused on characterizing the biological nature of those 88 repressed but ATRA/ATO-refractory genes. Gene ontology analysis indicated that 15 of them belonged to transcription factors or signal transducers (Figure 2d). Of note, these potential key regulators of myeloid cell proliferation and differentiation included several well-documented tumor suppressors such as *Irf8*, *Ldb1* and *Ikzf1*.^29, 30, 31^

### The repressed but ATRA/ATO-refractory genes in APL progenitors contain an Irf8-centered regulatory pathway of AML

**Figure 3:**
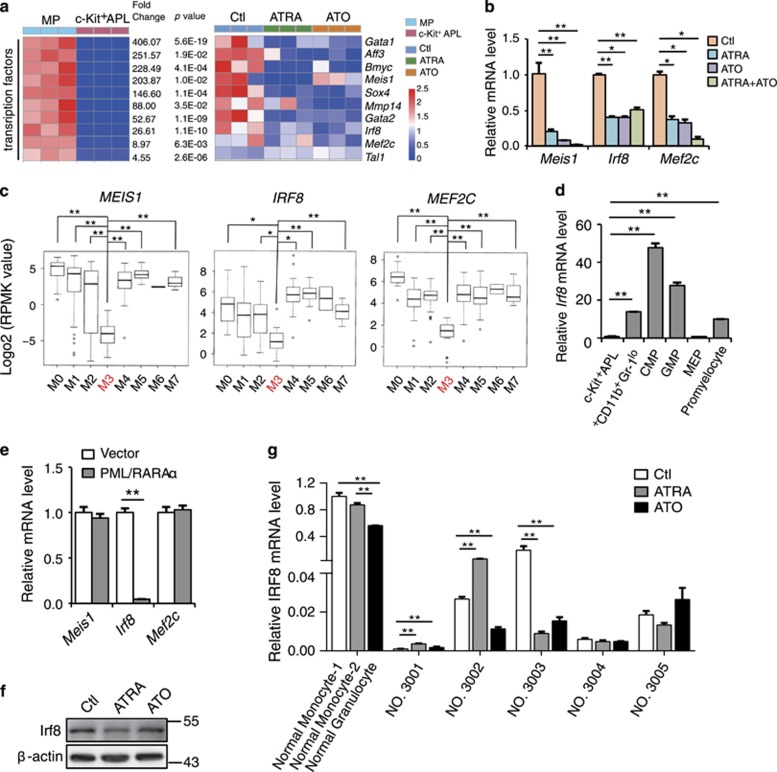
*Irf8* represents a PML/RAR*α*-target gene that is refractory to ATRA and ATO signaling. (**a**) Heatmaps of *Irf8*-centered dysregulated genes in c-Kit^+^ APL progenitors that were refractory to ATRA or ATO treatment. (**b**) Mouse APL cells were treated daily with ATRA or ATO alone or in combination for 6 days, and the mRNA expression levels of *Meis1*, *Irf8* and *Mef2c* were measured by RT-PCR. (**c**) The subtype-associated mRNA expression profiles of *MEIS1*,*IRF8* and *MEF2C* among 172 leukemic blast-enriched BM samples of human AML (FAB classification). The raw data were obtained from the The Cancer Genome Atlas AML database and were normalized to the *GAPDH* mRNA level. (**d**) Quantitative RT-PCR assay on the expression of *Irf8* in the c-Kit^+^ APL progenitors and normal myeloid subsets indicated. (**e**) PML/RAR*α* was transduced into normal c-Kit^+^ BM cells by retroviral infection, and the mRNA levels of *Meis1*, *Irf8* and *Mef2c* were measured by RT-PCR. (**f**) Western blot assay for the protein level of Irf8 in GFP^+^ mouse APL cells with or without ATRA or ATO treatment *in vivo*. (**g**) The RT-PCR measurement of *IRF8* mRNA levels in primary APL BM blasts treated with or without ATRA (1 *μ*M) or ATO (1 *μ*M) for 72 h *in vitro*. Normal monocytes and granulocytes were used as control. All data in this figure are presented as the mean±S.D., *P <0.05, **P<0.01

Then, we explored whether a potentially dysregulated myelopoiesis regulatory pathway was embedded within these repressed but ATRA/ATO signaling-refractory genes in the APL progenitors. Given the critical functions of transcription factors in normal or leukemic hematopoiesis, we addressed this issue by inspecting the transcription factors included in this gene pool, especially the top 10 most suppressed ones (Figure 3a) verified by semi-quantitative RT-PCR (Figure 3b; Supplementary Figures S2a and b). Interestingly, The Cancer Genome Atlas data indicated that the mRNA levels of *MEIS1*, *IRF8* and *MEF2C*, which constitute an IRF8-centered innate immunity pathway that suppressed the malignancy of AML cells,^29^ but not the other transcription factors, were lower in APL cases than in other subtypes of AML (Figure 3c; Supplementary Figure S2c). This result indicated that this IRF8-centered pathway was commonly dysregulated in human APL cases. In accordance, *Irf8* mRNA level was greatly decreased in the APL progenitors compared to all the potential cells of origin of APL including CMP, GMP, promyelocyte and immature c-Kit^+^CD11b^−^Gr^−^1^lo^ BM myeloid cells that immunophenotypically resembled c-Kit^+^ APL progenitors (Figure 3d).^32^ To test a possible repressing effect of PML/RAR*α* on *Irf8* expression, we transduced PML/RAR*α* into normal c-Kit^+^ BM cells via retrovirus infection, and revealed an immediate suppressive effect of PML/RAR*α* on the mRNA level of *Irf8* (Figure 3e). Nevertheless, we did not observe a similar effect on the mRNA levels of *MEIS1* and *MEF2C* that otherwise were not the direct target genes of PML/RAR*α*.^10^

Accordingly, Irf8 protein level in mouse APL cells remained unrestored after ATRA or ATO treatment (Figure 3f). To test the effect of ATRA or ATO on IRF8 expression in human APL cells, we measured the *IRF8* mRNA level in primary APL BM blasts. The *IRF8* mRNA levels were much lower in primary APL samples than in normal monocytes or even in neutrophils regardless of whether they were treated with or without ATRA or ATO (Figure 3g), indicating that the ATRA or ATO alone was insufficient to restore the depressed *IRF8* level in patient-derived APL cells.

### Irf8 expression increases with the spontaneous monocytic differentiation of APL progenitors

**Figure 4:**
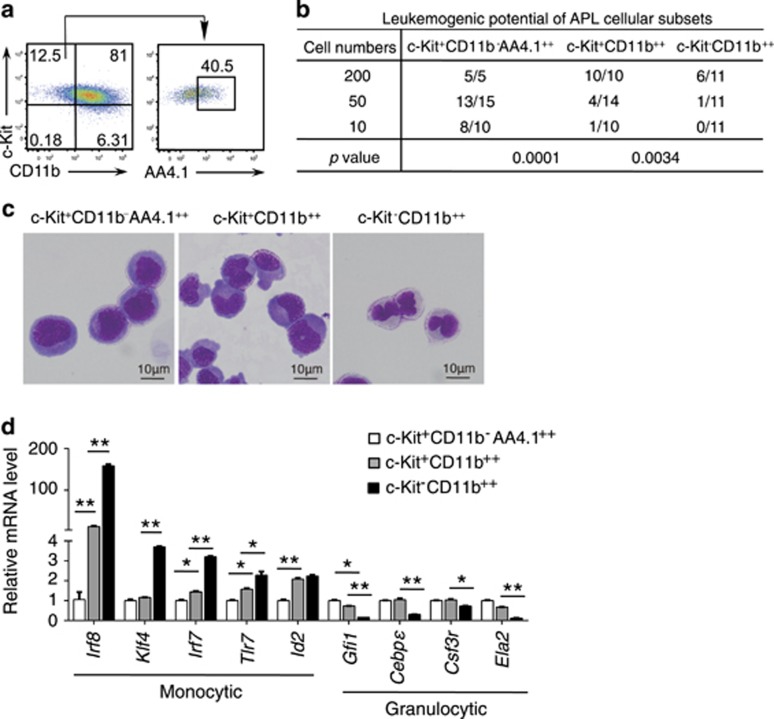
*Irf8* increases with the spontaneous monocytic differentiation of the APL progenitors. (**a**) Flow cytometry assay showing the differential expression of c-Kit, CD11b and AA4.1 in APL cells. (**b**) Measurement of the leukomogenic potential by inoculating serially diluted leukemia subsets into non-irradiated recipients. The observation time was ≥120 days. (**c**) Giemsa staining and microscopic inspection of sorted c-Kit^+^CD11b^−^ AA4.1^++^ cells, c-Kit^+^CD11b^++^ cells and c-Kit^−^CD11b^++^ cells in the mouse APL model. (**d**) The mRNA levels of monocytic and granulocytic maturation-related genes in the APL progenitors and c-Kit^−^CD11b^++^ mature cells in the mouse APL model as measured by RT-PCR assay. All data in this figure are presented as the mean±S.D., *P <0.05, **P<0.01

The leukemic cell population of AML is heterogeneous,^27, 33^ and it is well accepted that the leukemic cell-associated leukemogenic potential may decrease along with a likely spontaneous differentiation at a low rate. In line with this notion, cell-sorting and syngeneic transplantation experiments in the mouse APL model showed that the leukemia-initiating potential of APL cells declined step-wisely along with a potential myeloid differentiation-like process from c-Kit^+^CD11b^−^ AA4.1^++^ APL cells through c-Kit^+^CD11b^++^ APL cells towards c-Kit^-^CD11b^++^ APL cells (Figures 4a–c).

The leukemia progenitor cells in this APL mouse have been shown to differentiationally resemble normal GMPs with granulocytic and monocytic differentiation potentials.^24, 34^ To characterize the lineage directions of the spontaneous differentiation, we performed RT-PCR to measure the expressional alterations of the granulocytic or monocytic-specific transcription factors and others along with the transition form c-Kit^+^CD11b^-^ AA4.1^++^ APL cells through c-Kit^+^CD11b^++^ APL cells towards c-Kit^−^CD11b^++^ APL cells. Interestingly, the mRNA levels of monocytic rather than granulocytic differentiation-related transcription factors, including *Irf8* and *Klf4*, highly increased down this differentiation hierarchy, especially in the most mature c-Kit^−^CD11b^++^ APL cells (Figure 4d). These observations indicated the APL cells possessed a spontaneous monocytic differentiation potential *in vivo*. This prompted us to test whether derepressing the *Irf8* expression would diminish the leukemogenic potential of APL cells by driving them to undergo monocytic differentiation.

### Irf8 represses the establishment of the APL phenotype *in vivo*

**Figure 5:**
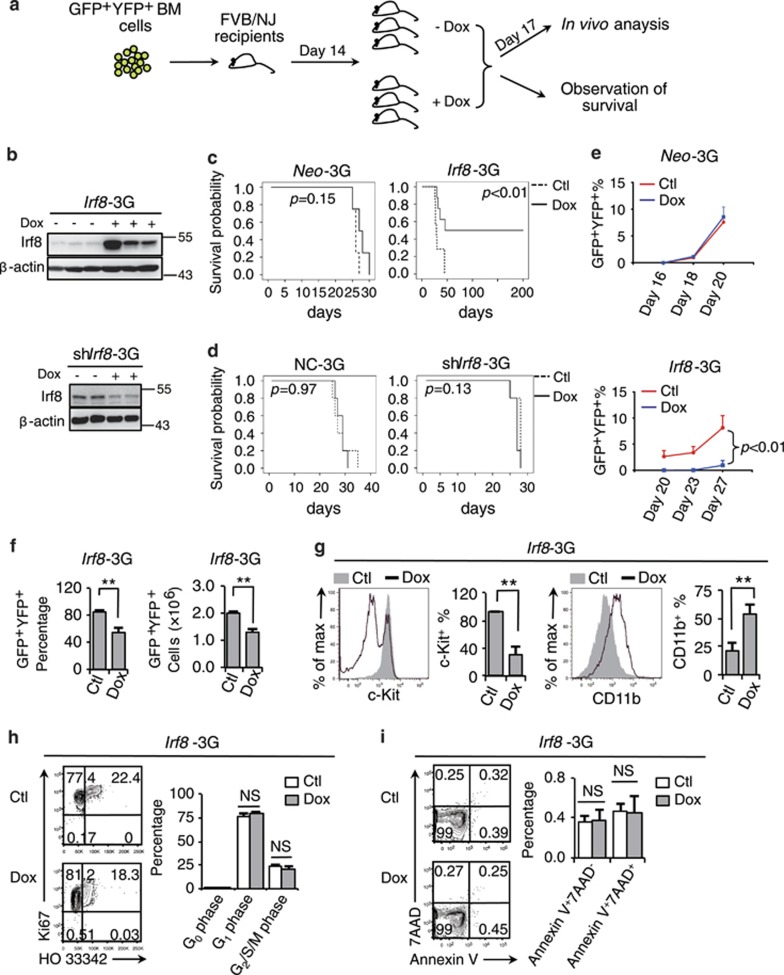
*Irf8* represents a potential oncorepressor in APL. (**a**) The experimental strategy used for the *in vivo* analyses of the APL mouse model genetically incorporated with a Tet-On3G gene-inducible expression. (**b**) Western blotting assay on the Irf8 protein level of APL cells (*Irf8*-3G, upper panel, or *shIrf8*-3G, bottom panel) with or without exposure to Dox administration (200 *μ*g/ml in the drinking water) for 3 days. (**c**–**d**) Kaplan–Meier survival curves of the *Irf8*-3G mice (**c**) and *shIrf8*-3G mice (**d**) after treatment with or without Dox (*n*≥5), *Neo*-3G (*n*=4) and NC-3G (*n*=5) mice were used as the controls. (**e**) Dynamic monitoring of GFP^+^YFP^+^ APL cell percentages in the peripheral blood of the *Neo*-3G or *Irf8*-3G mice after exposure to Dox. (**f**–**i**) The *Irf8*-3G mice were treated with or without Dox for 3 days. (**f**) The percentages (left panel) and absolute numbers (right panel) of GFP^+^YFP^+^ APL cells in the total BM cells. (**g**) The flow cytometry analyses of the expressions of c-Kit and CD11b within the leukemic BM compartment. (**h**–**i**) Flow cytometry analyses of the cell cycle (**h**) and survival (**i**) of BM APL cells. All data in this figure are presented as the mean±S.D., *P<0.05, **P<0.01

*Irf8* overexpression was recently shown to suppress leukemic proliferation in an experimental MN1/Meis1-overexpression mouse AML model.^29^ However, a clinical investigation of all subtypes of AML (150 cases) failed to reveal an expected reverse relationship between the *IRF8* mRNA level of leukemic blast BM samples and a poor prognosis (Supplementary Figure S3a).^35^ To test whether *Irf8* represented an authentic oncorepressor in APL, we tested the *in vitro* and *in vivo* effects of *Irf8* overexpression or knockdown on cell behaviors and malignancy maintenance of APL cells in an *Irf8*- or sh*Irf8*-inducible mouse APL model by doxycycline (Dox; Supplementary Figure S3b; Figures 5a and b). The administration of Dox itself did not exert obvious promoting or inhibitory effects on the establishment of leukemic phenotype, as evidenced by the similar survival curves observed for Neo-3G or NC-3G mice treated with or without Dox (Figures 5c and d). Nevertheless, the Dox-induced *Irf8* expression in the *Irf8*-3G model significantly inhibited the establishment of leukemic phenotype compared to the untreated *Irf8*-3G mice and Dox-treated Neo-3G mice (Figure 5c). The Dox-induced *Irf8* knockdown did not exert any obvious regulatory effect on the establishment of leukemic phenotype probably because that the Irf8 expression was already severely repressed in APL cells (Figure 5d). Furthermore, a 3-day administration of Dox did not influence the proliferation curves of APL cells in Neo-3G mice but did inhibit the proliferation of APL cells in *Irf8*-3G mice (Figure 5e), which was accompanied by a reduction of leukemia burden (Figure 5f), a promoted differentiation to c-Kit^+^CD11b^++^ mature APL cells (Figure 5g). Nevertheless, the reduced leukemia burden after *Irf8* induction was much less due to an inhibited cell cycle or a prompted apoptosis induction (Figures 5h and i). Likewise, a transient *Irf8* induction significantly promoted the myeloid differentiation of APL progenitors without exerting obvious regulatory effects on cell survival and proliferation *in vitro* (Supplementary Figures S3c–g). Taken together, these results indicate that *Irf8* acts as a potential oncorepressor of APL by driving leukemic progenitors to undergo myeloid differentiation.

### Irf8 overexpression unleashes the monocytic/dendritic cell differentiation potential of APL cells

APL LICs may developmentally correspond to multipotent MPs equal to or even above GMPs.^24, 25, 26^ Therefore, APL progenitors may at least possess a bipotent differentiation potential to both the granulocytic and monocytic lineages. In support of this, previous studies have shown that human APL progenitors bear monocytic differentiation potential in certain cases,^36, 37^ in addition to their well-documented granulocytic differentiation potential. Similarly, we observed that the expression of several genes that marked monocytic maturation, such as *Irf8*, *Klf4*, *Irf7* and *Tlr7*, and the expression of a few granulocytic genes were upregulated with the spontaneous maturation of the c-Kit^+^CD11b^++^ APL cells from c-Kit^+^ CD11b^+^ or cKit^+^CD11b^−^AA4.1^++^ APL progenitors (Figure 4d).

Previous studies have demonstrated two essential roles for *Irf8* in the regulation of normal myeloid differentiation: promoting the maturation of the monocytic lineage at the expense of granulocytic maturation from GMPs and the terminal maturation or survival of two types of dendritic cells (DCs).^28, 38, 39, 40^ Therefore, we hypothesized that *Irf8* repression served as a key PML/RAR*α*-driven oncogenic mechanism to restrict monocytic/dendritic differentiation leakage of the APL progenitors, which otherwise would erode their leukemogenic potential. Consistent with this notion, PML/RAR*α* overexpression inhibited *Irf8* induction, which was accompanied by DC differentiation retardation from the normal Lin^−^ immature hematopoietic cells (Supplementary Figures S4a and b).

**Figure 6:**
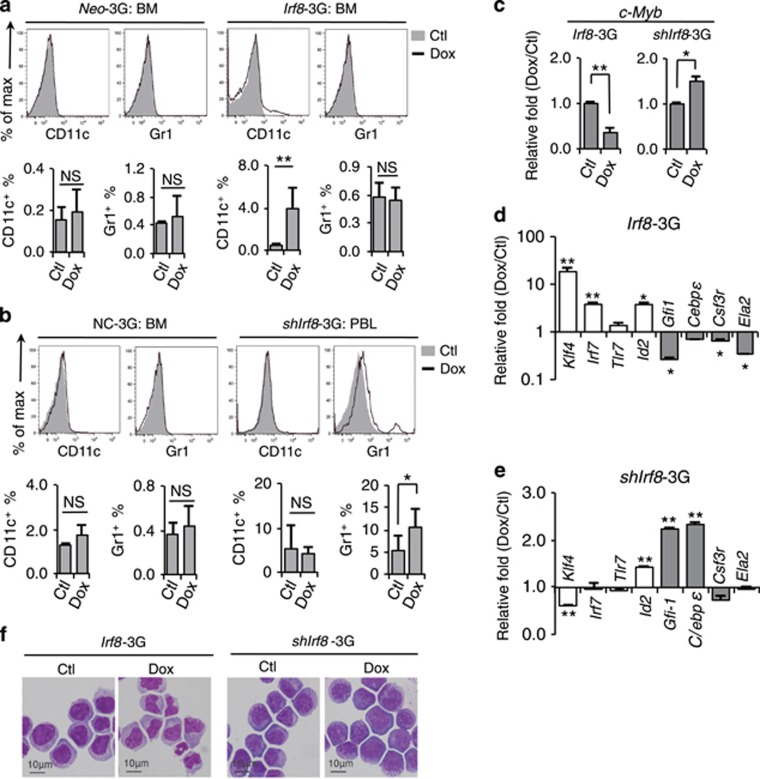
*Irf8* induction drives the monocytic/dendritic differentiation of APL progenitors. (**a**–**b**) Mice repopulated with *Neo*-3G or *Irf8*-3G APL cells (**a**) and with NC-3G or *shIrf8*-3G APL cells (**b**) were treated with Dox for 3 days *in vivo*. The percentages of CD11c^+^ and Gr-1^+^ cells within the leukemic cell compartment were analyzed by flow cytometry. The results of statistical analysis are shown in the bottom panels. (**c**) Quantitative RT-PCR assay on the stemness-related gene *c-Myb* in BM APL cells after *Irf8* overexpression or knockdown. (**d**–**e**) Quantitative RT-PCR assay on the monocytic (empty box) and granulocytic (filled box) differentiation-related genes in BM APL cells after *Irf8* overexpression (**d**) or knockdown (**e**). (**f**) Morphological inspection of *Irf8*-3G APL cells after *Irf8* induction (left panel) or knockdown *in vivo* (right panel). All data in this figure are presented as the mean±S.D., *P<0.05, **P<0.01

*Irf8* induction *in vivo* induced the generation of monocytic/dendritic CD11c^+^ cells, whereas *Irf8* knockdown increased the generation of granulocytic Gr-1^+^ cells from APL cells (Figures 6a and b). Analogous to this phenomenon, *Irf8* induction also promoted the monocytic/dendritic differentiation of APL cells *in vitro* (Supplementary Figures S4c and d). Furthermore, RT-PCR assay showed that *Irf8* induction promoted the expression of monocytic/dendritic differentiation-related genes and inhibited granulocytic differentiation and stemness maintenance-related gene expression, which was reversed to what was observed after *Irf8* knockdown (Figures 6c–e). *Irf8*-induced APL cells consistently displayed a monocytic rather than a granulocytic cell-like morphology, whereas APL cells in the sh*Irf8*-3G mice still morphologically resembled immature MPs (Figure 6f).

Nevertheless, *Irf8* induction did not alter PML/RAR*α* degradation and the PML nuclear body (NB) structure or increase p53 and p21 expressions, which was shown to be related to downstream pathways of ATRA and ATO (Supplementary Figures S4e–h). Moreover, *Irf8* knockdown but not *Irf8* induction moderately decreased *β*-catenin expression (Supplementary Figures S4g and h).

## Discussion

Although PML/RAR*α* has been shown to act via more than one route, the fusion protein transforms MPs and maintains their malignancy largely by interfering with the transcription of genes whose dynamic expression (regulated by physiological cues, such as RA) is required to execute stepwise and branching myeloid differentiations. In this study, we characterized how the transcription profile of APL cells was altered by ATRA or ATO administration *in vivo*. Similar to the data from the *in vitro* studies of human APL cell lines, our expression analyses indicated that ATRA specifically promoted granulocytic differentiation. Nevertheless, unlike what was suggested by the previous studies – ATO mainly induced apoptosis rather than myeloid differentiation of APL cells – we showed a granulocytic differentiation-promoting effect of ATO that was even stronger than the inducing effect of ATRA administration. The underlying reason for this discrepancy between *in vitro* and *in vivo* studies is currently unclear. However, it can be postulated that, although ATO induced a significant activation of p53 signaling pathway *in vivo*, a likely resultant apoptosis-inducing effect on APL cells could be counteracted by the supportive role of the remodeled hematopoietic microenvironment.^41^

Next, we analyzed whether ATRA or ATO corrected the expressions of all of the dysregulated genes that were potentially involved in the maintenance of immaturity and leukemogenic potential of the immature APL cells. Consistent with the recent observations revealing the differentiational heterogeneity of AML LSCs,^27^ our cell-sorting and transplantation experiments indicated that c-Kit^+^ APL cells, without or with an obvious CD11b expression, both retained substantial leukemogenic potential. Although the normal counterparts of these c-Kit^+^ APL cells within the normal hematopoietic hierarchy have remained unclear, accumulating evidences indicate that the APL LSC most likely originated from the malignant transformation of CMP and GMP, and to a lesser extent from MEP.^24, 25, 26^ Moreover, c-Kit^+^CD11b^+^ APL progenitors might developmentally resemble the unipotent monocytic or granulocytic progenitors or even promyelocytes. Therefore, we chose the normal Sca-1^−^Lin^−^ c-Kit^+^ MPs that contain CMPs, GMPs, MEPs, monocytic progenitor and granulocytic progenitors as the normal counterparts of APL progenitors.^28^ The comparison of APL progenitors and normal MPs produced a potential pool of dysregulated genes. To further enrich the real dysregulated genes in immature APL cells from this pool, we then overlapped these genes with the pool of PML/RAR*α* target genes.^10^ The observation that the expressions of only ~10% of these repressed genes were significantly reversed by ATRA or ATO indicated that ATRA or ATO was unable to correct all of the transcription repression resulting from PML/RAR*α*. Our results coincided with the previous observation that ATRA was able to correct the expression of ~10% of PML/RAR*α* target genes in human NB4 cells.^10^ Notably, an IRF8-centered regulatory pathway that has been shown to repress AML malignancy through upregulating innate immunity program was identified in this repressed but ATRA/ATO-refractory gene pool.^29^

Interestingly, we noticed that *Irf8* expression increased along with a likely spontaneous monocytic/dendritic differentiation of mouse APL cells *in vivo*, although the reasons behind this monocytic differentiation preference remain obscure and this spontaneous differentiation proceeded in quite a low rate. These observations are interesting within the context of the following three previous findings from other investigators: (1) the LSCs of APL probably differentiationally correspond to normal GMPs,^24, 34^ and therefore possess a bipotent differentiation potential towards granulocytic or monocytic direction; (2) monocytic differentiation can be induced in certain APL cases and also in human APL cell lines;^36, 37^ and (3) *Irf8* itself is a master gene that drives the monoytic differentiation of normal MPs and also promotes the maturation and survival of DCs.^28, 38, 39, 40^ Taken together, these observations suggest that the repressed *Irf8* expression stands as a potentially crucial mechanism for the maintenance of the immaturity and malignancy of APL LSCs by precluding a possible monocytic maturation leakage. This notion was supported by the Dox-induced expression and knockdown experiments of *Irf8* in APL cells, which showed a function role of *Irf8* to drive monocytic/dendritic differentiation at the expense of granulocytic differentiation, to be accompanied by a reduction in the leukemogenesis of APL cells. Why Irf8 knockdown did not significantly increase the leukemogenic potential of APL is current unclear. One possible explanation is that *Irf8* knockdown might potentiate the leukemogenic potential leakage through accelerating the granulocytic differentiation of immature APL cells. As *Irf8* induction drives a differentiation path of APL cells distinct from that by ATRA or ATO, it may serves an alternative differentiation induction strategy for those APL cases that are basically resistant to ATRA- or ATO-based therapies.

The reason why so many PML/RAR*α*-dysregulated genes do not respond to ATRA or ATO treatment despite evidence of the induced PML/RAR*α* degradation and granulocytic differentiation should be determined in the future work. For *Irf8*, we hypothesize that ATRA or ATO exerts dual roles in modulating the expression of *Irf8*. On one hand, the removal of PML/RAR*α* might derepress *Irf8* expression. On the other hand, the activation of granulocytic program may shut down the whole monocytic differentiation program including the induction of *Irf8* as a key event.

## Materials and methods

### Flow cytometry analysis and cell sorting

The flow cytometric data were collected on a BD (Becton Dickinson, Franklin Lakes, NJ, USA) Calibur or a LSRII flow cytometer and analyzed using FlowJo software (TreeStar, Ashland, OR, USA) or Summit software (Beckman Coulter, Fullerton, CA, USA). All antibodies were purchased from BD PharMingen or eBiosciences (CA, USA) as folollows: PE- or BV786- conjugated c-Kit, PE-Cy7-conjugated CD11b, PerCP-Cy5.5-conjugated Gr-1, APC-conjugated CD11c, APC-conjugated Annexin V and APC-conjugated Ki67. Total cells were Fc-blocked and stained with indicated combinations of antibodies for 30 min on ice, then washed three times and resuspended in 1% FBS/PBS. For apoptosis analysis, cells were resuspended with binding buffer and stained with Annexin V and 7AAD for 15 min at RT (25 °C) in the dark. For cell cycle analysis, cells were thoroughly resuspended and incubated with fixation and permeabilization solution for 20 min at room temperature, washed twice with BD Perm/Wash buffer and stained with HO33342 for 10 min. For cell sorting, the nucleated cells were stained with the indicated antibodies and resuspended in 2% FBS/PBS. The cells were sorted using a MoFlo machine (Beckman Coulter).

### Treatment of leukemia with ATRA, ATO and Dox

Both ATRA treatment (10 mg/kg) and ATO (ATO, 4 mg/kg, Sigma) were administrated by daily intraperitoneal injection of the same amount of ATRA dissolved in DMSO for the indicated times. Dox (200 g/ml) was administrated in the drinking water that was changed every 3 days.

### Analysis of RNA deep-sequencing data

Raw sequence reads were initially processed using FastQC (Babraham Institute, Cambridge, UK) for quality control, and then adapter sequences and poor quality reads were removed using Cutadapt. Quality-filtered reads were then mapped to mm9 using STAR, and only uniquely mapped reads were kept. Read counts were calculated using HTSeq-count. Differentially expressed genes were identified using R package DESeq2 (*P*<0.05, fold change >1.5). All the RNA-seq raw data have been deposited in GEO database under the accession numbers GSE46434 and GSE94017.

### Statistical analyses

Kaplan–Meier software (SPSS 16.0, Chicago, IL, USA) was used to analyze the survival probabilities of different animal groups. Other results were analyzed using the *t*-test and are expressed as the mean±S.D. Values with *P*<0.05 were considered significant, *P<0.05, **P<0.01.
