# Supplementary material for: All-trans retinoic acid and arsenic trioxide fail to derepress the monocytic differentiation driver Irf8 in acute promyelocytic leukemia cells
Source: Cell Death Dis. 2017 May 11;8(5):e2782–. doi: 10.1038/cddis.2017.197 (PMC5520717; doi:10.1038/cddis.2017.197)
Supplement: Supplementary Material [file cddis2017197x1.docx]

**Supplementary Material:**

1. **Supplementary figures and figure legends:**
2. Supplementary Figure 1, related to Figure1. GFP labeling of APL cells and their response to ATRA or ATO treatment.
3. Supplementary Figure 2, related to Figure 3. Irf8 is a refractory PML-RARα target gene to ATRA or ATO**.**
4. Supplementary Figure 3, related to Figure 5. Irf8 as an oncorepressor of APL cells.
5. Supplementary Figure 4, related to Figure 6. Irf8 induces the monocytic/dendritic differentiation of APL cells.
6. **Supplementary Material and Methods.**

**Supplementary Figure Legends:**

**Supplementary Figure 1. GFP labeling of APL cells and their responses to ATRA or ATO treatment, related to Figure 1.**

(a) Wright-Giemsa staining and flow cytometric analyses of APL BM cells before and after GFP labeling by retroviral transduction of MigR1 vector. (b-d) Representative flow cytometric analyses of the c-Kit and CD11b, Gr1 and CD11c expressions to assess myeloid differentiation (b), Annexin V and 7AAD staining to assess cell survival (c), and HO33342 and Ki67 staining to assess cell cycle (d). (e) KEGG pathway enrichment assay of DE genes in mouse APL cells altered by ATRA or ATO *in vivo*.


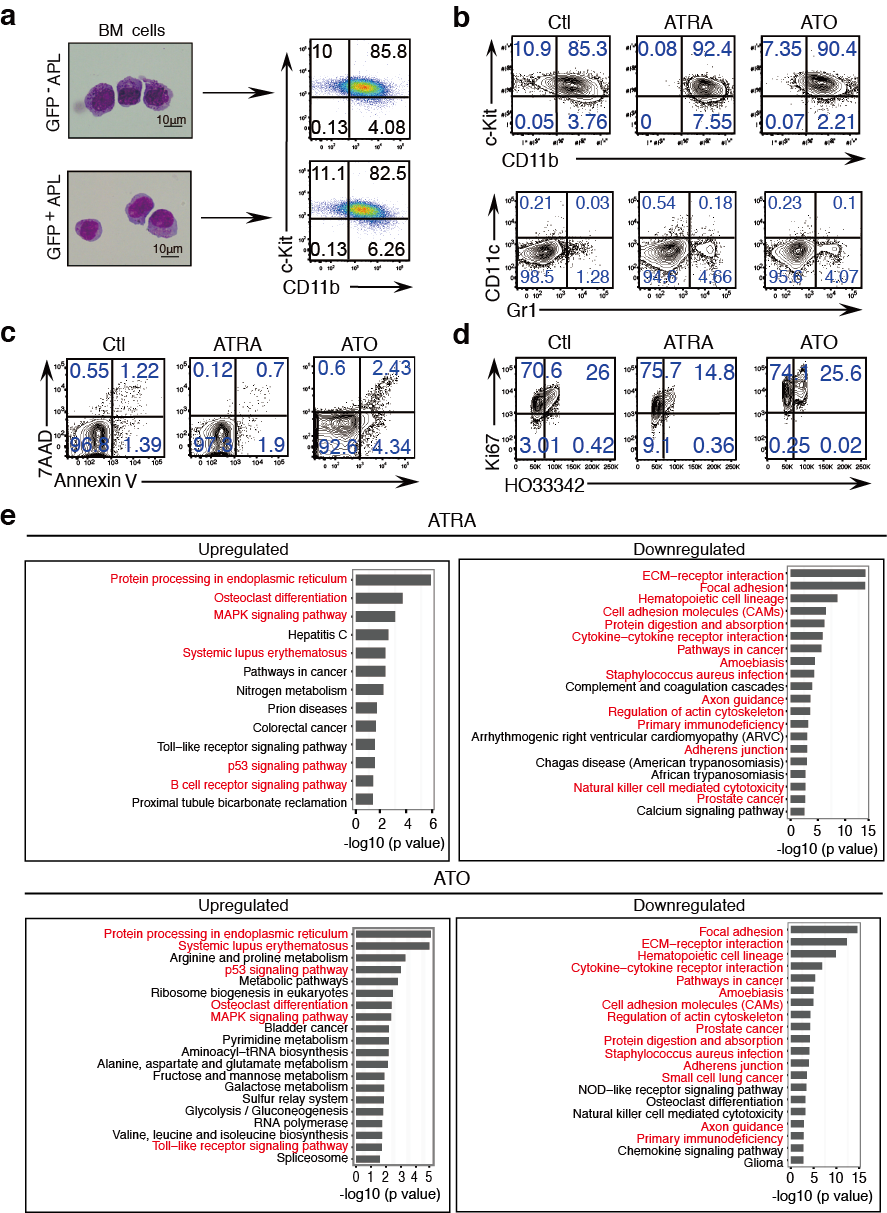


**Supplementary Figure 2. Irf8 is a PML/RARα target gene that is refractory to ATRA or ATO, related to Figure 3.**

(a) Quantitative RT-PCR verification of the downregulated genes in the c-Kit^+^ APL progenitors that were refractory to ATRA or ATO treatment. (b) Quantitative RT-PCR verification of the *Gata1, Aff3, Bmyc, Sox4, Mmp14, Gata2* and *Tal1* mRNA levels post-ATRA or ATO administration alone or in a combination. (c) The *GATA1, AFF3, SOX4, MMP14, GATA2* and *TAL1* mRNA expression levels among 8 different AML subtypes. All data in this figure are presented as the mean ± SD, * p <0.05, ** p<0.01.


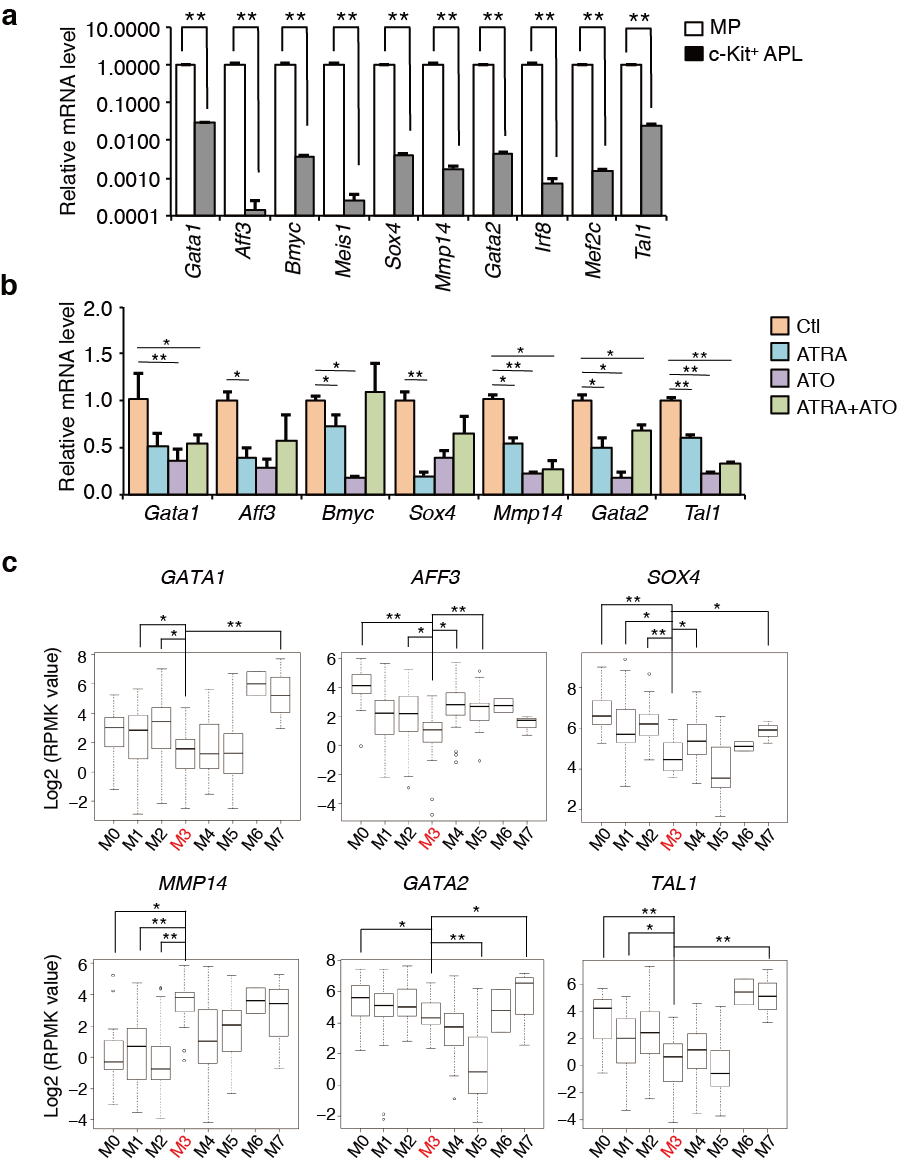


**Supplementary Figure 3. Irf8 as an oncorepressor of APL cells, related to Figure 5.**

(a) Survival curves of AML patients with high *IRF8* mRNA level (blue) and low *IRF8* mRNA level (red). The raw data were obtained from the TCGA database, and analyzed using the OncoLne software. (b) Flow chart for the incorporation of a Dox-inducible expression system into mouse APL cells, namely Irf8-3G or Neo-3G APL cells. The diagrams for the retroviral vectors are shown in the upper panel. (c-g) Primary GFP^+^YFP^+^ BM APL cells isolated from Irf8-3G or Neo-3G mice were treated with PBS or 1 μg/ml Dox for the indicated times and cultured *in vitro*. (c) Ectopic expression of Irf8 was verified by western blotting after the addition of Dox. Proliferation curves (d), c-Kit or CD11b expression (e), cell survival (f) and the cell cycle status (g) were monitored by flow cytometry. All data in this figure are presented as the mean±SD, * p <0.05, ** p<0.01.


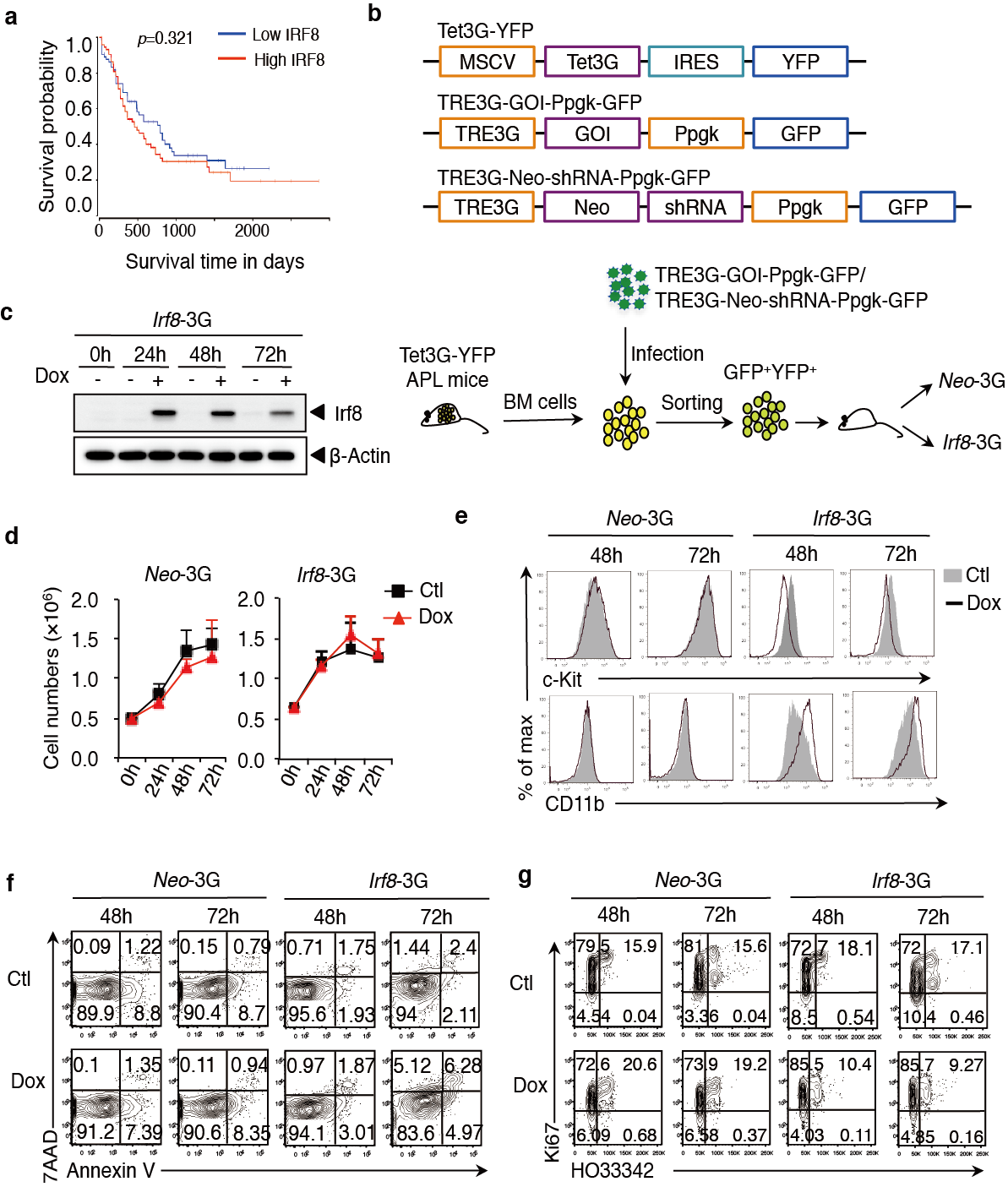


**Supplementary Figure 4. Irf8 induces the monocytic/dendritic differentiation of APL cells, related to Figure 6.**

(a-b) Lin^-^ BM cells were transduced by empty vector or PML/RARα-MigR1 and cultured in dendritic cell induction medium. The mRNA level of *Irf8* expression measured by quantitative RT-PCR (a) and the production of CD11c^+^ dendritic cells assessed by flow cytometric analysis (b) are shown. (c-d) Primary BM *Neo*-3G or *Irf8*-3G APL cells were treated with PBS or 1 μg/ml Dox for the indicated times and cultured *in vitro*. (c) Wright-Giemsa staining of the *Irf8*-3G APL cells treated with or without Dox. (d) The expressions of the myeloid differentiation antigens CD11c/Gr-1/CD115/F4/80 were measured by flow cytometry. (e) The protein level of PML/RARα was measured in APL cells isolated from *Irf8*-3G mice with or without Dox treatment *in vivo* for 6 days. (f) Immunofluorescence detection of PML nuclear bodies within APL leukemia cells after ATRA treatment or *Irf8* up-regulation. The cells were stained with DAPI and a Cy3-conjugated PML antibody. (g-h) Quantitative RT-PCR assay on the *β-catenin*, *p53* and *p21* in BM APL cells after ectopic *Irf8* induction (g) or knockdown (h). All data in this figure are presented as the mean±SD, * p <0.05, ** p<0.01.

**
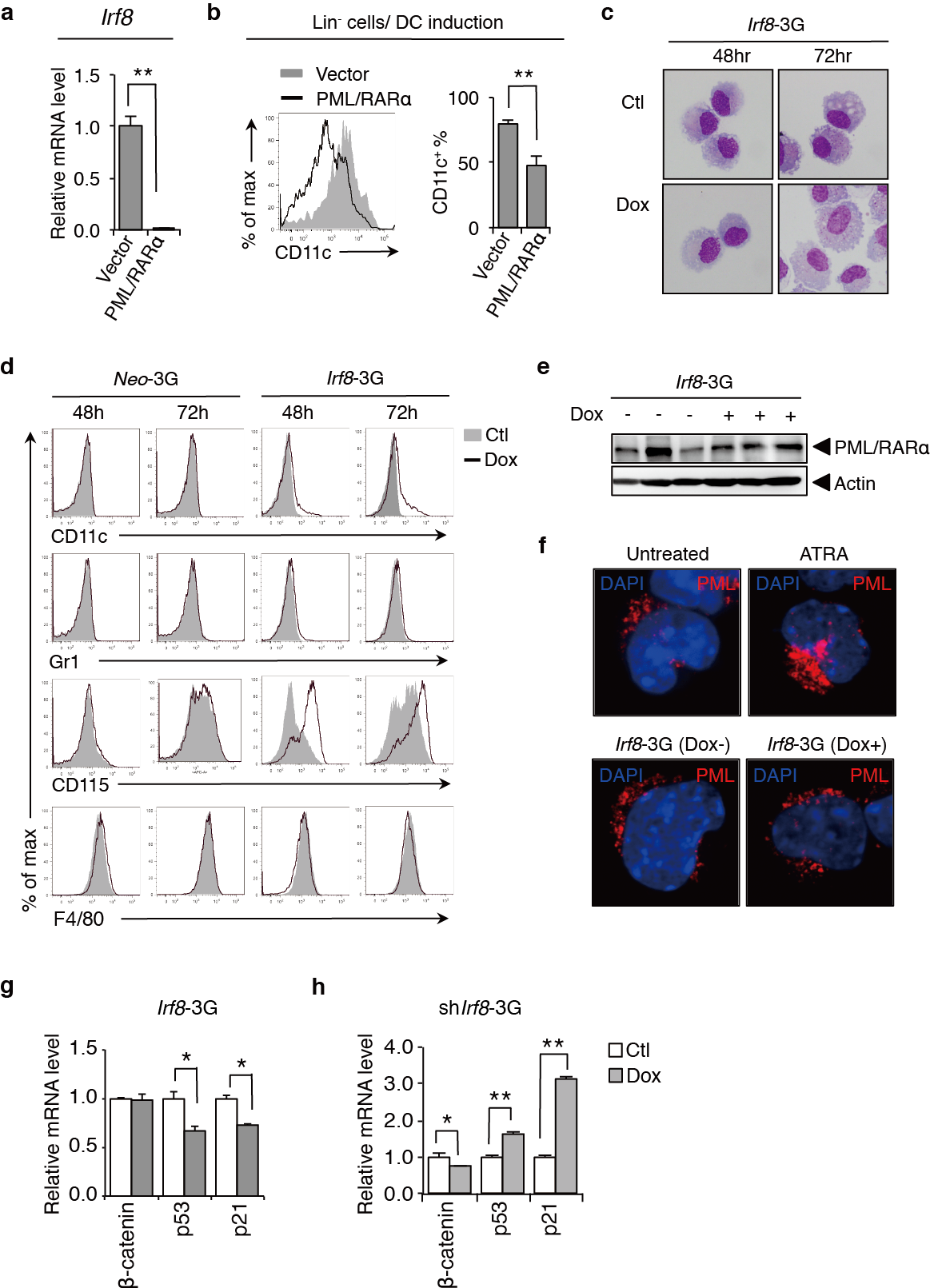
**

**Supplementary Material and Methods:**

**Leukemia cell transplantation**

In all transplantation experiments performed, mouse APL-like cells or their subsets were intravenously injected into non-irradiated or sublethally-irradiated syngeneic mice aged between 6 to 10 weeks. In cases where the number of injected APL-like cells was ≤10, the leukemia cells were mixed with 1×10^5^ normal spleen cells prior to injection.

**Limiting Dilution Analysis**

The sorted leukemic subsets were transplanted into recipients after limited dilution, and then the survival status of recipients was monitored. The LIC frequencies of indicated leukemic subsets were calculated by L-Calc^TM^ Limiting Dilution software (Stem Cell^TM^ Technologies).

**GFP labeling and genetic incorporation of a Dox-inducible expression system into leukemia cells by retroviral transduction**

The retroviral plasmid MigR1 and the packaging plasmid Ecopac were co-transfected into 293T cells to produce the retroviral supernatant. In total, 5-10×10^5^ c-Kit^+^CD11b^-^ BM cells from moribund APL mice were incubated with the retroviral supernatant in RPMI 1640 media containing 10% FBS, 50 ng/ml mSCF, 10 ng/ml mIL-6, 6 ng/ml mIL-3 (R&D) and 4 μg/ml polybrene (Sigma) for 48 hours. The cells were harvested and sorted for the GFP^+^ subpopulation, which was then transplanted into sub-lethally irradiated (450 cGy) FVB/NJ mice. After four to five weeks, the recipients were sacrificed when they became moribund, and the labeled primary GFP^+^ leukemia subsets were harvested. For Dox inducible expression systems, the plasmids Tet3G-MigR1-YFP or pRetro-TRE3G-GFP were transfected into plate E cells to package the retrovirus. The APL cells were then infected with these retroviruses sequentially, and GFP^+^YFP^+^ leukemia BM cells were sorted out for further study.

**Quantitative real-time RT-PCR and western blotting analyses**

Total cellular RNA was extracted using the RNeasy micro kit or RNeasy mini kit (QIAGEN, Valencia, CA) following the manufacturer’s protocol. The cDNA was synthesized through reverse transcription of 1 μg of RNA using ReverTra Ace-α^TM^ (TOYOBO, Kita-ku, Osaka, Japan). Quantitative real-time RT-PCR reactions were performed using SYBR Premix Ex Taq (Applied Takara Bio Inc.) on an ABI 7300 Real-Time PCR system. The primer sequences for the detection of target genes are acquired from online: <https://pga.mgh.harvard.edu/primerbank/>. Western blotting was performed using standard protocols.

**Wright-Giemsa, POX and DCE stainings**

Sorted cells were cytospun onto slides and stained with Wright-Giemsa staining solution following manufacturer’s protocol. Peroxidase and D chloroacetate esterase staining was performed using a cytochemistry staining kit (Sunbio, Shanghai, China). The samples were observed under a light microscope (DP51, Olympus).
